# National role conceptions and populist parties in Europe between heterogeneity and convergence

**DOI:** 10.1057/s41295-022-00325-1

**Published:** 2022-11-15

**Authors:** Pietro de Perini

**Affiliations:** Department of Political Science, Law and International Studies, University of Padova, Via del Santo, 28, 35123 Padova, Italy

**Keywords:** Foreign policy, Populism, National role conceptions, Discourse, Rassemblement National, Podemos, Five star movement

## Abstract

Drawing from the National Role Conceptions (NRCs) framework, this paper seeks to assess whether, beneath the ideological, structural or discursive differences exposed in the literature, a pattern of convergence among the foreign policies of populist parties in Europe can be identified in how these conceive the orientation and tasks that their countries should follow in the international system. Comparing the NRCs which emerge from the foreign policy discourse of three populist parties of diverse persuasions—*Front National/Rassemblement National* in France, *Movimento 5 Stelle* in Italy, *Podemos* in Spain—the paper argues that these parties share the underlying conception of a decisively pro-active, transformative and value-based role for their countries in both European and global perspectives, which the different articulations of the people/other dichotomy in their foreign policy discourse affect and differentiate in terms of goals and preferences.

.

## Introduction

As populists have emerged throughout Europe as ‘serious contenders for power’ (Font et al. 2021, p. 164), their increased participation in parliaments and governments has also meant a greater interest in the foreign policy agendas of their countries, requiring populist parties to develop their own international priorities and objectives. A recent but fast-growing body of scholarship is investigating this process, with particular attention to its effects on foreign policy choice and implementation in the countries where populists have reached a position of power.

Most scholars have addressed the specific behaviour of one populist party or leader, typically in one country or on specific foreign policy areas (Cadier 2021; Coticchia 2021; Verbeek and Zaslove 2014; Destradi et al. 2021; Giurlando 2020; Exadaktylos 2020). Others have investigated commonalities and differences among ideologically like-minded populists, especially within the radical right (Kane and McCulloch 2017; Falk and Stahl 2022). Some others have analysed the positions of populist parties with respect to different international cooperative orders, for instance, in the context of EU foreign policy (Balfour et al. 2016; Cadier and Lequesne 2020) or in relation to Atlanticism (Chryssogelos 2021).

Whether the focus is on Europe or on other areas, the literature makes a general claim for the heterogeneity of populist foreign policy. Such heterogeneity has been explained with reference to either the different thicker host ideologies of analysed parties (Verbeek and Zaslove 2017; Chryssogelos 2017; Wehner and Thies 2020), the specific structural features or strategic cultures of the states where these operate (Chryssogelos 2021), the policy paradigms to which they refer (Exadaktylos 2020), or the diverse discursive strategies applied by their leaders (Wojczewski 2020). The common conclusion that these studies have reached starting from different conceptual and empirical perspectives implicitly suggests that there may not be any distinctive characteristics about populist foreign policy-making at all (Chryssogelos 2021).

Drawing from Role Theory, and specifically from the National Role Conceptions (NRCs) framework, this paper aims to investigate whether a pattern of convergence can be found among the scopes of foreign policy behaviours that the leaders of diverse populist parties consider as appropriate for their states to undertake, namely comparing how these leaders conceive and present the general tasks and roles that their countries should perform in the broader international system.

At their roots, role-theoretical approaches to foreign policy analysis posit that a state’s decisions and actions are consistent with policymakers’ conceptions of their nation’s orientations and tasks in the international and regional systems, referred to as NRCs (Holsti 1970, pp. 244–245; Thies 2010). In addition to being the first step in trying to explain, and even predict, a given country’s foreign policy choice and implementation (Wish 1980; Le Prestre 1997), identifying existing NRCs represents a promising strategy to understand how the preferences and ideas of political leaders combine with considerations of national material and cultural resources and capabilities in a ‘narration’ that reproduces the expected foreign policy posture these conceive for their country (Isernia and Longo 2017; Wehner 2020). In this case, the focus of analysis can be moved from foreign policy implementation to foreign policy discourse and be applied regardless of the position that the leaders under consideration play in national governments. Indeed, while role scholarship has traditionally focused on decision-makers’ conceptions and assessed their actual performance, scholars engaged in this field have also insisted on the importance of considering how other domestic actors within the political spectrum develop and present their own role conceptions (Cantir and Kaarbo 2012, 2016).

Consistent with this view, this paper explores possible convergences among populist foreign policy conceptions in Europe by investigating and comparing the NRCs that emerge from the discourse of populist parties of different persuasions. In particular, the analysis is conducted on three parties: *Front National/Rassemblement National* (FN/RN) in France, *Movimento 5 Stelle* (M5S) in Italy, *Podemos* in Spain.

Applying the NRCs framework to the analysis of the discourse of selected parties, this paper seeks to set the stage for an original conceptual and analytical perspective to grasp similarities and differences between a variety of populist expressions in Europe, including those who have not fulfilled significant governmental responsibilities. In addition, it expects to contribute to more systematically relating insights among the external orientations of diverse populist parties in the continent, and to complement recent attempts to explain and compare populist foreign policy at both global (Friedrichs 2022; Frahm and Lehmkuhl 2022) and regional levels (Destradi et al. 2021, p. 665; Falk and Stahl 2022: Burrier 2019; Wehner and Thies 2020; Wajner 2019).

The first section of this paper discusses the key debates surrounding the nexus between foreign policy and populism in scholarship, and introduces the original conceptual contribution brought by the introduction of the NRC framework. The second section elaborates on the analytical framework and methodological approach adopted. The third and last section addresses the common pattern identified by the NRCs shared by all selected parties and discusses why and how this underlying area of convergence often translates in different articulations of foreign policy preferences and goals. The conclusions summarize the key conceptual and empirical contributions of this paper and discuss additional research paths to consolidate this approach.

## Conceptual framework: foreign policy, populism and the benefits of NRCs

### Conceptualizing the relationship between populism and foreign policy

Unlike Latin America, where populism is a long-established, diffuse and dynamic phenomenon which has evolved through different phases and approaches—from its ‘classical’ protectionist and politically inclusive version in the 1930s, to the nationalist, regional integrationist and state interventionist wave of ‘Bolivarian populism’ since the late 1990s (Burrier 2019)—in Europe, this phenomenon stood up more recently. Although they certainly did not emerge from nowhere (Jones 2007; Verbeek and Zaslove, 2014), populist parties have spread and consolidated throughout the continent especially during the 2010s, a period where ‘political analysts began to refer to the “comeback” of populism with increasing intensity, and IR scholars posited that contemporary politics was entering an era of global populism’ (Wajner 2019, 196). This ‘Europe’s populist surge’ (Mudde 2016) gave rise to heterogeneous antagonistic responses in many countries to protect the ‘people’ facing a sustained period of multidimensional crises (Caiani and Graziano 2019), particularly marked by those of the Eurozone and refugees (Balfour et al. 2016).

Despite the remarkable academic attention raised by the consolidation of this phenomenon, foreign policy making of European populists has not been an immediate concern. Scholars have motivated their late interest by claiming that, in addition to migration and European integration, populist parties have developed little interest in wider international issues and thus have lagged behind in developing their own priorities and goals in this sector (Chryssogelos 2021; Balfour et al. 2016). Foreign policy is also generally considered a distant and elite-driven process where ‘institutional memory’ and long-career civil servants play a prominent role (Hill 2016), clashing with populists’ generalized scepticism and even opposition to elite, however elite is constructed (Destradi et al. 2021). Eventually, while in all its past and current expressions populism can be considered first and foremost a domestic process that *can have* international ramifications (Wehner and Thies 2020, 7, italics added), the increasing threats brought to national and popular sovereignty by international politics and globalization have prompted populists to progressively commit themselves to shape their country’s foreign policy orientation and actions (Verbeek and Zaslove 2014, 526).

One of the few substantive points of agreement in the specialized literature is that ‘populist foreign policies are diverse and not monolithic’ (Wehner and Thies 2020, p. 16). This is a fundamental finding whether sets of populist foreign policies are investigated in the current European stream (Balfour et al 2016; Falk and Stahl 2022), in Latin America, where populism is, as mentioned, historically established and more homogenous across the diverse phases of its evolution (Hawkins et al. 2019; Wajner 2019), or are addressed globally across leaders with similar ideological or cultural perspectives (Frahm and Lehmkuhl 2022).

However, the explanation for this heterogeneity changes significantly depending on how populist foreign policy is conceptually approached. Focusing on the structural level of analysis, for example, Chryssogelos (2021) considers that populist foreign action in Europe reflects mainly the influence of established strategic traditions of the countries with which they operate rather than populist ideology per se. Ideational approaches, however, are prevalent, and often explain heterogeneity in foreign-policy making pointing to the prior host ideology to which populism is attached (Destradi et al. 2021), that is, the ‘ideological bedfellow’ which steers populists’ positions regarding international challenges and, hence, their foreign policy preferences (Verbeek and Zaslove 2017, p. 398).

Some scholars, in fact, have recognized in peculiar experiences, such as in the ‘neo-imperialism populism’ of autocratic parties in Turkey and Russia, a specific and genuine ‘thick’ ideologic nature (Frahm and Lehmkuhl 2022). However, the majority of studies that focus on ideational factors consider populism as a ‘thin centred’ ideology, that is, a set of ideas that is limited in ambition and scope (Mudde 2004) and, therefore, do not provide fully articulated and coherent perspectives regarding the social and political world (Verbeek and Zaslove 2014). This scholarship recognizes three main concepts that characterize the thin core of populism: the pure people, the corrupt elite (which are constructed in a Manichean relationship), and the general will to which populists appeal, that is, ‘the idea that all individuals of a given community are able to unify their wills with the aim of proclaiming popular sovereignty as the only legitimate source of political power’ (Mudde and Rovira Kaltwasser 2013b, p. 151).

Foreign policy analysts working on this track have generally distinguished populist parties and their international agency across the traditional left/right cleavage. Populist parties of the right show foreign policy positions that reflect their nativism, opposition to immigration, and rejection of economic and cultural globalization; populist parties of the left reject neoliberalism and open markets (Chryssogelos 2017; Falk and Stahl 2022). Despite this apparently neat distinction, populist parties proved to have unpredictable positions on a wide range of international matters, making their outcomes remain uncertain and ambivalent even if they relate to the same host ideology (Destradi et al 2021; Verbeek and Zaslove 2014). Furthermore, not all populist parties can be easily located in one of these two categories as many populists remain ideologically ambiguous (Burrier 2019, p. 169).

An alternative approach to disentangle populist varieties differentiates between ‘exclusionary’ and ‘inclusionary’ (Mudde and Rovira Kaltwasser 2013a). In this case, a key factor is the way these actors define ‘the others’ (Caiani and Graziano 2019). This approach to classification appears suitable to capture the ambiguity of populist parties’ foreign policy preferences, especially as far as the ‘symbolic dimension’ of this dichotomy is concerned, which inquires on how the boundaries between the ‘people’ and the ‘elite’ (or, more broadly, ‘the others’) are set in political discourse (Mudde and Rovira Kaltwasser 2013b).

On one hand, investigating who or what is included or excluded from these core categories promises to better capture populists’ understanding of international socialization processes and the types of cooperation they pursue transversally toward the external world. On the other, since to some extent the inclusionary/exclusionary dichotomy contains the left/right one (Caiani and Graziano 2019), this approach both connects with the literature employing the ideational approach, and complements insights from another relevant strand of research: that which understands populist foreign policy primarily through discourse (Wojczewski 2020; Kriesi and Pappas 2015).

Some consider discourse in alternative to ideology, as just ‘rhetoric’, which reduces populism to ‘a new way of talking about foreign policy while doing largely the same things’ (Chryssogelos 2021, 18). Consistent with this view, many studies concerned with populist foreign policy implementation have found little shifts in external decision-making when these parties reach power (Verbeek and Zaslove 2017; Plagemann and Destradi 2019). Other discursive-related approaches, however, place their focus on how ‘the populist notion of the people can also be a subjectivity that is constituted and reproduced via the discourse of foreign policy’ (Wojczewski 2020, 294). From this perspective, focusing on discourse allows an original perspective to understand the rationale of populist’s understanding of foreign policy, which can also be understood as a strategy of identity formation of ‘the people’ through ‘negative Othering’ (Friedrichs 2022). According to how this dichotomy is developed by the populist elite, the foreign other can include, vertically, a globalist establishment or a multilateral organization and, horizontally, the elites of international adversaries or, if the ethnocultural perspective of many exclusionary parties in Europe is considered, specific social groups, including migrants, members of minorities, and religious communities.

If populist foreign policy is understood as the ‘extension of the pure people interest’ (Wicaksana 2022), investigating how the populist core categories can be placed in different antagonistic relationships in the realm of foreign policy also allows tracking ‘a set of different discursive strategies that distinguish among foreign policies orientations, and, arguably, practices’ (Wojczewski 2020, p. 294; see also Exadaktylos 2020, p. 187). After all, as Hall (2021) notes, while the notion of who are the people is often implicit and vague, ‘who is outside this group is normally made clear via the rhetorical demonization of outsiders’. Similarly to what the symbolic dimension of the inclusionary/exclusionary framework entails, therefore, the emphasis in the analysis of discursive-related approaches is on how foreign policy contents are articulated through a distinct discourse, or ‘narration’ (Wehner 2020), which reproduces how people and others are constructed and related in foreign policy making (Wojczewski 2020, p. 298).

Although eventually acknowledging the diversity of foreign policy rhetoric and implementation, all of these studies also make important steps forward in 'tidying' this complex, ambiguous, and inconsistent area of investigation, reducing the observed heterogeneity of foreign policy of populist parties to a limited set of strategies or discursive approaches, which in some cases transversely hold populists of different persuasions and geographic areas. For instance, a recent study by Friedrichs (2022), who focuses on the ‘national identity conceptions’ of individual populist leaders across the word, demonstrates the importance of the emotional correlates of fear and pride in constructing all populist foreign policy’s preferences analysed.

Role theory promises a further complementary step in efforts looking at common patterns and convergence in populist foreign policy discourse. Exposing how populist parties conceive and present their expectations about the tasks and functions of their state within the international system as a whole, NRCs offer an alternative and little explored perspective to grasp the construction and presentation of populist foreign policy preferences and an additional level of analysis which integrates and complements the key insights of the above-discussed approaches.

### The benefits of Role Theory and NRCs

Part of the explanatory value of Role Theory is recognized in its capacity to adapt and be incorporated into other conceptual approaches (Wehner 2020). As roles are generally viewed as ‘sets of expectations about the proper behaviour of an actor in a given social position’ (Mc Court 2011, p. 1607), their applications to foreign policy analysis traditionally understand key decisions and actions as resulting from, or being consistent with, policymakers' conceptions of their nation's orientations and tasks in the international system or in subordinate regional systems (Holsti 1970, pp. 244–245; Thies and Breuning 2012). Consequently, NRCs—that is, policy makers' own definitions of the general kind of decisions, commitments, rules and functions, their state should carry out continuously in the international system (Holsti 1970, pp. 245–246)—allow delineating the scope of foreign policy behaviours that decision makers can imagine and perceive as appropriate for their states to undertake (Breuning 2011, p. 23).

NRCs do not emerge exclusively out of one ‘actor’s own consideration of its place, position and appropriate behaviour vis-à-vis others in a given social environment (‘ego’ part of the role); they can be also affected by the expectations of other actors (‘alter’ part), which can be either achieved, ascribed or cast in the broader process of international socialization (Isernia and Longo 2017, p. 112; Harnisch et al. 2011). In general, therefore, the identification of a role demonstrates particularities that stem ‘both from what the type of actors’ ego and alter are, and from their respective cultural repertoires as attitudes and actions needed to both resonate with domestic audience and be accepted by international others’ (Wehner 2020). Consequently, as Breuning (2011, p. 26) points out, the NRC framework ‘seeks to understand how actors fashion their role in the international system, navigating between domestic sources of identity and/or cultural heritage, taking advantage of the material resources at their disposal, circumnavigating as best as possible the obstacles imposed by their position in the international structure’. While the possibility of understanding foreign policy implementation (‘role enactment’ in role theory jargon), strongly relies on an integration of self-conceptions and external expectations (Nabers 2011), the specific focus of this article on how populist parties understand, imagine and ultimately present their countries’ desired international orientations prioritizes analysis on the ego part of their NRCs focusing primarily on their foreign policy discourse.

A significant body of role-theoretical foreign policy analysis concentrates on the conceptions of decision-makers in charge of their country’s government (Le Prestre 1997; Wish 1980). Rulers are considered to be in the best position to more directly and effectively shape and try to perform internationally their conceptions. However, scholars engaged in the development of role theory have insisted on the opportunity to consider the ways in which all actors within the domestic political spectrum, including opposition and public opinion, develop their own NRC, as these could even affect the definition of the country's ego conceptions through ‘contestation’ (Cantir and Kaarbo 2016). This approach entails opening the ‘black box’ of national politics and focusing on the conceptions held by specific groups and single individuals (Cantir and Karboo 2012). Thus, isolating populist parties’ role conceptions can be analytically meaningful per se, regardless of the different levels of access that populist parties have had to national power, especially since the bulk of this analysis lies in their conceptions in foreign policy discourse, not on agency, decision-making or implementation.

While confirming findings on populist foreign policy heterogeneity, the study by Wehner and Thies (2020) on Argentina and Venezuela, one of the few applying role theory to the subject matter, claims that the key difference between a populist and a non-populist foreign policy is the rhetoric used to justify role choice. Indeed, NRCs are built to a large extent from material and cultural considerations about the status, opportunities and constraints of a country in the wider international system, as they are perceived and presented by a given leadership (Chafetz et al. 1996). Leadership itself depends both on understanding the culture and identity of a national society and on translating both into a role conception that resonates domestically (Hudson 1999). In presenting foreign policy priorities and approaches, therefore, populists are also advancing ‘a narration of the type of the international actor they want their state to be by following principles and casting roles that (would) help them to achieve their foreign policy goals’ (Wehner and Thies 2020, p. 7).

However, there is not necessarily a single way to think of a specific role. Actors can interpret and narrate the same NRCs differently, consistent with their political ambitions, geographical position, and cultural and ideational considerations. This implies that populist parties can share the same underlying orientation in international politics but articulate it through different preferences and goals, including with respect to how exclusionary or inclusionary the ‘people’ and 'others' are constructed and narrated in their foreign policy discourse.

Therefore, the NRCs framework complements and innovates the current debate on similarities and differences among populist foreign policies. At one level, looking at how NRCs are presented and articulated in discourse provides an original view on how the leaders of populist parties transform their understanding of national cultural heritage, material resources, and international opportunities and constraints into a general foreign policy narration. This level of analysis overlaps with and intersects referred discursive approaches and the ‘symbolic’ dimension of the inclusionary/exclusionary framework, lingering on the importance of shaping the boundaries between ‘us’ (the people) and ‘the others’ in the construction of NRCs. In addition to these considerations—prior to them, in fact—role theory allows grasping how these parties understand the broader functioning of the system of international relations and how they believe their state could have a say to make it less hostile to their ‘people’, however inclusionary or exclusionary these are defined.

### Analytical framework and methodology

In light of the conceptual crossroad discussed above, the symbolic dimension of the inclusionary/exclusionary classification, represents a promising standpoint to search for any convergence in the role conceptions among the diversity of European populists. Similar to left/right, however, this classification is based on a binary distinction, which does not allow satisfactorily positioning ‘contradictory, ambiguous, and opportunistic’ populists on the ideological spectrum (Burrier 2019, p. 166), and only encompasses what Zulianello (2020) defines as ‘positional populists’. To ensure a richer heterogeneity of case-selection, therefore, this paper refers to a ‘gradational approach’ that considers ‘exclusionary’ and ‘inclusionary’ as two ends within a continuum (Font et al. 2021), within which diverse populist parties can be positioned with a certain degree of flexibility.

Consistent with this analytical strategy, this paper investigates the foreign policy discourse of three populist parties: FN/RN in France, M5S in Italy, and *Podemos* in Spain. All three parties are widely recognized as ‘populist’ in the literature and have acquired a consolidated position in their national political scenario. While the Italian and Spanish parties were established more recently and directly contributed to shape the alleged ‘era of global populism’ during the 2010s (Balfour et al. 2016), the French one, created in 1972, was in fact a precursor of this phenomenon (Surel 2019). However, FN/RN has increased its popularity and renovated its international visibility following the change of leadership between Jean-Marie and Marine Le Pen in 2011, also benefiting from the improved communication skills of the incumbent leader (Williams 2011). Also in light of the circumscribed time-frame of this study (2017–2019, see further below), this permits to analytically considering FN/RN together with Podemos and the M5S as three diverse expressions of the same recent European populist surge.

In particular, the three parties are located at different points within the inclusionary/exclusionary spectrum. The FN/RN, as many other European radical right and xenophobic populist parties, is positioned around the exclusionary end (Surel 2019) and *Podemos* around the inclusionary one (Font et al. 2021). Analyses of the M5S highlight a more inconsistent positioning (Coticchia 2021; Font et al. 2021), making it a particularly interesting addition to case selection. On one hand, the M5S shares some preferences with current inclusionary parties in Europe and Latin America especially in terms of opposing international economic elites and supporting (sub)regional integration to protect a largely constructed conception of ‘the people’ (Fossati 2020; Wajner 2019). On the other, the Italian party shares with the radical right an exclusionary approach, especially vis-à-vis migration (Diodato 2022).

The current case-selection aims therefore to capture the ‘chameleonic nature’ of populism (Hall 2021, p. 51) offering a ‘useful variation of the dimensions of the theoretical interest’ (see Falk and Stahl 2022). There are other as successful populist parties in Europe across the inclusionary/exclusionary continuum which could have been considered for this analysis (Alternative for Germany, Fidesz or Syriza, for instance). However, the selection has also sought to restrict the area of reference—focusing only on southwestern Europe—in order to limit as much as possible the salience of structural and contextual geographical, historical and cultural factors and their impact on role shaping, thus making the 'role sets' of the selected parties easier to compare and discuss.

Although the FN/RN has always been among the opposition, despite a growing success among the French electorate, Podemos has been attributed two ministries in the current Spanish government, while, since 2018, the M5S has had major government responsibilities (it played a key role in the Conte and Draghi cabinets, and also expressed the Ministry of Foreign Affairs). As this difference in the access to power may appear problematic from an analytical perspective, it is important to reiterate that the goal of this paper is not to assess the impact of these parties on national foreign policy-making and implementation. The analysis is limited to how role conceptions are expressed in policy discourse, regardless of how they are affected in practice by domestic and international opportunities and pre-existing diplomatic structures (Hawkins et al 2019; Friedrichs 2022; de Perini 2021). From this perspective, what is analytically crucial is that these three parties realistically competed for the leadership of their countries in the period under analysis, causing them to develop and present a full-fledged political agenda, including on the whole range of international affairs.

Notwithstanding, these three parties have not articulated an as clear and thorough vision on foreign policy matters. *Podemos*, as other inclusionary populist parties in Europe and Latin America is strongly internationalist. This emerges frequently in the party’s discourse, as when it explains that ‘faced with the crisis of multilateralism, our country must lead the commitment to advance international democracy’ (Podemos 2019, p. 57). *Podemos* has a very elaborate and consistent agenda and its own International Relations Secretary, which has been responsible for much of the party’s agenda-setting since the party’s establishment in 2014 (Real Instituto Elcano 2015). International issues represent a constant underlying element of FN/RN priorities too, especially in an anti-terrorism and anti-immigration perspective (Kane and McCulloch 2017) although the party has not established any dedicated foreign policy office, unit, or directorate (Camus 2016). The M5S has generally provided a little defined, and thus vague foreign policy agenda (Cadier and Lequesne 2020). A 10-point foreign affairs programme ('*Programma Esteri*') has been presented in the run-up to 2018 political elections to its militants who voted online for these points on a priority list (M5S 2018a; Di Stefano 2017). However, as Diodato (2022, 10) underlines, for the M5s ‘topics on foreign policy are not believed to be good for the people whether the people want them or not’. Similarly to the FN/RN, there is no formal unit or post for international affairs within the M5S organigram.

These different degrees of development and articulation of foreign policy goals, orientations, and infrastructure entail that the NRCs which emerge from discourse are either very well defined and consolidated (*Podemos*), well defined but eventually subsumed into the broader political agenda and thus more difficult to isolate (FN/RN), or little elaborated and mostly rendered via slogans (M5S). However, this diversity in both form and substance does not preclude capturing the overall international posturing and task that each of these parties conceives and expresses for their country as they emerge in relation to identified NRCs, without yearning to cover all of the foreign policy priorities and objectives of these parties.

Holsti (1970, p. 260) introduced 17 NRCs. However, new roles are constantly being conceived, especially through a subjective reinterpretation of the past of analysed countries (Wehner 2020). Scholars have applied and revised this initial list, occasionally adding new roles and discarding others that had become archaic (Caffarena and Gabusi 2017; Oppermann 2012; Gurol and Starkmann 2021; Thies and Wehner 2020). Therefore, the number of identified NRCs, some of which sometimes overlap, is not definite. Among the various NRCs that can be extracted from these parties’ narration, this paper discusses only those shared by the three of them, identifying an underlying area of convergence that the different articulations of the people/other dichotomy in the discourse of each party affect and differentiate in terms of goals and preferences.

NRCs are assigned through a qualitative content analysis of selected populist party policy documents (Neuendorf 2004; Wesley 2014). The bulk of the analysis is on the three parties’ electoral manifestos, which, in light of the research design of this paper, present a number of advantages. First, they operate as points of reference to examine how the parties defined their positions on national and foreign policy issues as presented to voters, providing, thus, a direct measure of the ideas of politicians as they are communicated to the public (Hawkins et al. 2019; Exadaktylos 2020), In addition, for all three parties, manifestos are presented in a very direct style, which replicates and condenses the discourse of their leaders but, more effectively than speeches or interviews, facilitate a sufficiently reliable comparison between the three parties. Manifestos also present the most systematic discussion of the views and expectations of these parties and guarantee that, even when perceived as niche elements, foreign policy issues are adequately dealt with for all selected parties. Manifestos also represent the synthesis of the political debate between parties’ leaders and voters. Therefore, they contain the final result of internal role-shaping processes, where possible divergences and contestations between populist leaders or between them and their ‘base’ are eventually reunited. With a view to both supply where manifestos are not sufficiently elaborated and triangulate findings, the paper refers to additional documents, including speeches by the leading foreign policy figures of selected parties, parties' documents on important issues, such as environment and migration, parliamentary bills and transcripts.

As the positioning of populist parties may be inconsistent and change overtime in light of specific interests, constraints and opportunities (Burrier 2019), the time frame in reference is, as mentioned, particularly circumscribed. It revolves around the latest completed national elections for each country surrounding the 2019 European elections, which were common to all. This analytical strategy seeks to include the two most significant electoral events with implications for foreign policy while reducing external variation as much as possible. While 2019 marks the end point of this time span for all parties, the other cut-off point changes from country to country. The French presidential elections considered are those of 2017, since those of 2022 were held in a completely different international milieu characterized by Covid-19 and the war in Ukraine. Italy had its latest political elections in 2018. Spain experienced, in fact, two general elections in 2019, in April and November, which surrounded the European election campaign.

Qualitative content analysis moves into the middle ground between the positivistic, systematic and quantitative features of traditional content analysis and more interpretative ‘discourse analysis’, within a wider ‘discourse analytic’ perspective (Hardy et al. 2004). By establishing a relationship between different elements, discourse ‘constitutes the meanings of subjects, objects, and practices and thereby provides a particular way of interpreting and understanding social reality’ (Wojczewski 2020, p. 294). This implies that in formally reproducing a discourse also all textual analysis eventually becomes an exercise in interpretation which allows examining what the messages provided by selected contents convey, given their context and circumstances (Herman 2008).

One or more NRCs are manually assigned to retrieved segments from the three parties’ electoral manifestos using the themes and labels from the literature. The focus is primarily on claims and sentences that convey the desired international posture of these parties expressing cultural or history-based considerations, which, in addition to being a starting point for defining NRCs, are also key factors in enhancing populism (Laclau 2005). Selected segments of text thus result in attributing a set of NRCs to each party that expresses them. When the same NRCs are attributed to the three of them, a convergence among their general foreign policy understanding is expected. Since the original sources are in French, Spanish or Italian, to ensure overall readability while revealing ‘discourse’, a selection of meaningful segments of text is literally translated into English by the author.

## Shared NRCs between convergence and differences

Content analysis results in the identification of 12 different NRCs from the manifestos of the three selected parties. Each NRC is assigned a label, which is exemplified by a set of ‘themes’, as summarized in reference Table 1.

**Table 1:** List and attribution of the NRCs extrapolated from the manifestos of FR/RN, Podemos and M5S

	NRCs*	Themes	FN/RN	Podemos	M5S
1	Regional leader	Duties or special responsibilities that an actor perceives for itself in its relation to states in a particular region with which it identifies	X	X	X
2	Liberation supporter	Unstructured and vague attitudes about actions required for organizing, leading, or physically supporting liberation movements abroad		X	X
3	Anti-imperialist agent	Perceptions as agent of ‘struggle’ against the threats of imperialism	X	X	X
4	Defender of the peace	statements that seem to indicate a universal commitment to defend against any aggression or threat to peace, no matter what the locale		X	X
5	Cultural power	Culture as a significant tool to be deployed to raise the country’s standing in the world, since it represents a respected source of contribution to the legacy of the humankind	X		
6	Developer	Perceptions of special duty or obligation to assist underdeveloped countries	X	X	X
7	Defender of the Faith	View of foreign policy objectives and commitments in terms of defending value systems from attack	X (as Defender of secularism, from Islamists)		
8	Effective multilateralist/ responsible state	Seeing multilateralism as a powerful tool for responsible states to make global governance work,			X
9	Principled actor	Belief that whatever decision, action, or behaviour becomes an option, an evaluation of their compatibility with an actor’s defining values	X	X	X
10	Independent	Emphasize elements of policy self-determination; no particular continuing task or function in the system	X		X
11	Example	Importance of promoting prestige and gaining influence in the international system by pursuing certain domestic policies	X	X	X
12	Internal development	Indication of a wish to remain non-involved in international political matters, but the statements do not preclude various forms of international cooperation, particularly in economic and technical matter	X		

*NRCs 1, 2, 3, 4, 6, 7, 10, 11, 12 have been introduced by Holsti (1970); NRCs 5, 8, 9 by Caffarena and Gabusi (2017)

A few of these NRCs were assigned only to one party. These include the RN/FN conceptions of ‘internal developer’—which is expressed primarily from an ecological perspective (Rassemblement National 2019a; 2021)—and ‘cultural power’, particularly evident in the repeated references to the hegemonic role of French language, culture, and heritage in shaping the discourse of the party regarding Europe, Africa and its overseas territories (Rassemblement National 2019b, 3; 2019c; Le Pen et al. 2018, p. 9). Other NRCs are shared by two parties. For instance, that of ‘liberation supporter’, which *Podemos* expresses explicitly with reference to the liberation of Western Sahara, Palestine, Kurdistan and Columbia (Podemos 2019, 57; 2016, 49; 2018, 6 and 11), and the M5S more implicitly, stating its intention to work for the recognition of Palestine ‘within the borders established by the UN in 1967’ (*sic*) (M5S 2018a) and through its generalized attempt at infusing ‘global governance with the demands of the unrepresented people’ (Diodato 2022).

As Table 1 shows, five of the overall 12 identified NRCs—those of ‘regional leader’, ‘example’, ‘anti-imperialist agent’, ‘developer’ and ‘principled actor’—have been attributed to all three parties. This paper claims that the co-occurrence of these five NRCs—regrouped into two distinct 'subsets', labelled 'transformative agent' and ‘value advocate’—outlines an underlying pattern of convergence among the general foreign policy orientations of these parties, which is now analysed and discussed in its diverse discursive articulations.

### ‘Transformative agent’

This role-subset combines three NRCs: ‘regional leader’, ‘example’, and ‘anti-imperialist agent’. The three parties express their NRC 'regional leader' primarily with regard to shaping the future of Europe, with some fundamental differences in how the NRC is articulated. In the conception of FN/RN, this role is conceived in revolutionary and antagonistic terms and revolves around the goal of establishing and leading a ‘European Alliance of Nations’, based on cooperation among singular national identities protected by certain external borders, instead of the current European Union (Rassemblement National 2019a, 2019b). Realizing this objective ‘is a special responsibility as French people, heirs of the first European state to have found its political form’ (Rassemblement National 2019b, 20). Part of this special duty is expressed through a renewed political vision to manage French overseas territories, which in the conception of the party would represent a backup of both French and European maritime power versus non-European competitors (Rassemblement National 2019c). The NRC of ‘regional leader’ for FN/RN lies, therefore, in a plan to give Europe the ‘stable and definite political form without which Europe will not exist’ (Rassemblement National 2019b, p. 17) and in the idea of ‘refunding Europe to conciliate it with its peoples’ (Rassemblement National 2019b, p. 19; Le Pen 2017a). Exclusionary lines, however, define this project which is meant to engage only a limited number of European nations and peoples: ‘geography, history, civilization decide.[…] This conviction gives all its coherence, its strength, its historical significance to this French path for a new cooperation in Europe’ (Rassemblement National 2019b, p. 19). The FN/RN conception for Europe is eventually that of ‘an alliance of sovereign nations defined by singular identities; that, above all, of a territory protected by an external border’ (idem, 17).

*Podemos* and the M5S also refer to the leading role that their countries should play in prompting a significant shift in the EU. The change they envision is as much antagonistic in tone and mainly revolves around the need to cancel austerity measures and reform economic governance to adapt it to the needs of the different national economies (Podemos 2016, p. 3; M5S 2018a). Consistent with its inclusionary character, Podemos aims to replace the current system with an ‘economic model that puts the interests of the social majority before those of the privileged, the banks and the multinationals’ (Podemos 2017). Furthermore, it expresses its main task for Spain by the will of 'recovering the idea of a democratic Europe, which is a space of human rights, freedoms, peace and cooperation in the face of this austerity Europe that today is advancing towards the abyss of xenophobia, social exclusion, and inequality’ (Podemos 2017). The ‘regional leader’ role of the M5S is, in fact, a ‘sub-regional leader’ role when Europe is concerned. This conception is built on the idea of ‘us’ as encompassing the south of Europe and is based on the conviction that ‘to save Europe, the countries of the South of the continent should, in the shortest possible time, form a common front that knows how to pose a credible ultimatum in Brussels’ (M5S 2018a).

A complementary articulation of the concept of 'regional leader' of M5S surfaces with respect to the Mediterranean region, where the leading role of Italy is expressed in regard to antiterrorism, the ‘immediate cessation of military intervention disguised as “humanitarian”, and disarmament’ (M5S 2018a). A leadership role concerning the Mediterranean emerges as well in *Podemos*’ conception, which is mostly expressed in efforts at reinforcing further the Spanish maritime rescue service and supporting NGOs to save migrants from this area (Podemos 2019, 63). While a general attention on the Mediterranean is not surprising, given that the paper’s case selection includes only parties from Southwestern Europe, the little reference that the FN/RN places on the role of France in such region in unanticipated. In the 2017 presidential election programme, expressed through 144 commitments, none of these referred to the Mediterranean or individual countries of that region (Le Pen 2017a), while in the party manifesto for European elections, the region is occasionally mentioned as a source of challenges, without any reference to the leading role that France should play there (Rassemblement National 2019b, p. 53).

The ‘example’ NRC outlines a more passive international engagement, since the main focus is on domestic policies that may bring prestige and leadership (Holsti 1970, p. 268). In addition to conceiving itself as a role model in saving human lives in the Mediterranean, *Podemos* also conceives this role with regard to other policy areas, including environment and climate-induced migration (Podemos 2016, p. 17; 2018, p. 14). This role is largely articulated in the party’s inclusionary understanding of society (that is, the ‘people’): ‘we work to build an international democratic alliance that defends popular sovereignty, justice and brotherhood between peoples, and we defend that our country can and must be a reference in the struggle to achieve a more just, more democratic, better world’ (Podemos 2018, p. 5).

Ecology and the environment are also one of the key elements that characterize the ‘example’ conception of FN/RN for France, especially considering the model the country should represent in the revolutionized idea of Europe of the party. In fact, based on the French natural heritage and its commitment to preserve it, its project ‘will help the Nations by all means to make Europe the first ecological civilization in the world and to give progress its true meaning: a better life for all’ (Rassemblement National 2019b, p. 62). Another policy area for which the FN/RN conceives France as an example, both regionally and globally, is that of migration. In their discourse, the exclusionary approach that the party pursues nationally should indeed aim for the political stability of various countries to avoid forced flows of populations fleeing the war and to prevent destabilized countries from becoming themselves sieve countries, controlled by the smugglers (Le Pen et al. 2018, p. 8). Their planned policy for overseas territories is expected to, among other objectives, ‘regain a space of security in the face of migratory attacks’ (Rassemblement National 2019c).

The ‘example’ role of the M5S is much less elaborated and mainly revolves around the parading of the global ‘excellences’ of the country’s cultural heritage, biodiversity and of the ‘Made in Italy’ (M5S 2018a, 2018b, p. 3; 2019) presenting, similar to the FN/RN, a mainly nationalistic construction of the people it aims to represent and protect.

The third NRC that contributes to shaping the ‘transformative agent’ role-subset is that of ‘anti-imperialist agent’, with ‘imperialism’ here broadly understood as the extension or imposition of power, authority, or influence by ‘predatory elites’ (see Destradi et al. 2021). All the parties perceive the primary vector of this imperialism in supranational European institutions, especially in terms of economic and financial governance. From this perspective the NRC of ‘anti-imperialist agent’ qualifies that of ‘regional leader’ in all three cases.

More specifically, in the FN/RN conception, the entire manifesto for the 2019 European elections is developed to expose France’s leading role in reversing an idea of Europe, whose ‘decision-making process is flawed’ and whose orientation is imposed in the most complete opacity by lobbyists and pressure groups in the service of multinationals, categorical or community interests (Rassemblement National 2019a). The European Commission is conceived as the key agent of this imposition also for its ‘punishing countries when they exercise their national sovereignty and refuse the political guidelines of Brussels’ (Rassemblement National 2019a, 4). In the FN/RN conception, in other words, ‘the EU has become the worst enemy of Europe and of Europeans’ (Rassemblement National 2019b, p. 13). European institutions, indeed, show not only to ‘know that they are governing against the people, but above all, that they are ready to use all legal tricks or pressure to prevent the people from deciding’ (Rassemblement National 2019a, 5). France, therefore, should ‘start the fight to free Europe from any dependence on systems, organizations, financial commitments that subject it to choices that are not its own’ (Rassemblement National 2019b, p. 30). Besides Europe, this NRCs for FN/RN is expressed also with regards to other ‘imperialist’ threats, such as those led from major maritime powers, such as the US and China (Rassemblement National 2019c).

The ‘anti-imperialist agent’ conception for *Podemos* also refers primarily to the EU. However, it is more narrowly articulated, as it focuses only on how financial and migration policies of the Union should be countered: ‘we do not recognize ourselves in the Europe of austerity, which sees inequality, poverty, xenophobia and racism grow hand in hand. That Europe is not worth us and we want to transform it’ (Real Instituto Elcano 2015). Austerity measures and their implications on national sovereignty remain, therefore, the key imposition from outside that Spain’s agency should counter. In *Podemos* conception, indeed, ‘austerity threatens our democracies: we want an economic model that puts the interests of the social majorities before those of the privileged, the banks and the multinationals’ (Podemos 2017; 2019). Thus, although the EU remains part of the puzzle, Spain’s agency against ‘financial imperialism’ and its institutions expands far beyond European borders (Podemos 2019, p. 55; Podemos 2016, p. 16) and tries to protect a much wider conception of people than that of the FN/RN.

As expected from the findings that see the M5S closer to inclusionary and left-leaning populist parties with regard to economic foreign policy (Chryssogelos 2021; Fossati 2020), the ‘anti-imperialist agent’ NRC of *Podemos* is more or less replicated in the conception of the Italian party. It is expressed, for instance, through the intentions of safeguarding the quality of Italian products from external influence and unfair competition and opposing ‘“in every way” all those blackmails of the markets and international finance disguised as “reforms”’(M5S 2018b, p. 3; 2019). The key imperialist agents that Italy should dismantle are then the European Mechanism of Stability and the so-called ‘Troika’, perceived as ‘supranational bodies that have contracted out people's democracy by imposing, without any popular mandate, the notorious “rigorous conditionalities”’. Even in this area, the M5S conceives Italy’s agency to fight these practices jointly with that of other Southern European states’ (M5S 2018a). In both parties, therefore, the anti-imperialist agent NRC identifies the traditional anti-globalization perspective of left-leaning populist parties which can be traced back to the earlier waves of populism in Latin America (Burrier 2019). Still commentators do not assess this similarity as enough to justify placing the M5S with Podemos (and other southern European countries’ parties, as Syriza in Greece), into the same family of inclusionary populist mobilization (Diodato 2022, p. 9; Font et al. 2021).

### ‘Value advocate’

The second role-subset points out that the overall foreign policy posture conceived by these three parties reflects a moral commitment to promote and protect defining principles and values besides material interests. The NRC 'principled actor', while attributed to all, develops significant differences that substantially depend on the principles and values that each party establishes at the roots of its international action and feels the responsibility to promote for protecting the people, and which, accordingly, diverge between more inclusionary (or universalist) and more exclusionary.

Chryssogelos (2017) claims that ‘“sovereignty” is probably the term that most accurately captures the populist logic of international affairs’. In fact, an exaltation of sovereignty emerges from the discourse of all selected parties. The protection of sovereignty is central in motivating the opposition to both international and European economic governance and is therefore the *raison d'etre* of the interest of these parties in external matters. However, sovereignty is rarely left alone. More frequently, it is paired with other values which these parties seek to advance as a key driver of their international agency. For *Podemos*, Spain should build an ‘international democratic alliance that in addition to popular sovereignty, defends justice and brotherhood among peoples’ (Podemos 2018, p. 5). FN/RN reminds that the sovereignty of states is inseparable from the principles and rules of international law (Le Pen 2017b). For FN/RN the protection of sovereignty is therefore the cornerstone of any form of international cooperation, including its development policy toward Africa (Le Pen et al. 2018, 9). For the M5S, the protection of national sovereignty is also the founding principle for the foreign policy of the party. This is complemented by other, at times contradictorily expressed, principles such as people’s self-determination, territorial integrity, non-interference in the internal affairs of individual countries, respect for dialogue between peoples, and the rigorous application of the principles enshrined in the UN Charter, which also include the promotion of universal human rights. However, the latter principles rank lower than ‘protect sovereignty’ in the preferences of the militants expressed through online voting (M5S 2018a). Multilateralism emerges as well as a key value of international agency. However, as Diodato (2022) underlines, this is intended in a more exclusionary fashion, as a new alternative form of cooperation based on a wider multilateralism of people built around the Mediterranean basin. Although it recalls the 'genetic multilateralism' of Italy (Andreatta 2008), this conception is distinctively populist as it includes a central reference to exalting the ‘people’ and an antagonistic discursive logic (Diodato 2022).

The way in which these values and principles are defined and presented by the M5S is just sketched in comparison to the thorough and universalistic conception of *Podemos*, for which, ‘the key principles of democracy, human rights and gender equality, and sustainable and equitable development will inform the whole of the foreign action of our government’ (Podemos 2019, pp. 56–57) and shall ‘prevail over geopolitical and economic interests and over certain security logics’ (Real Instituto Elcano 2015, p. 20), also through the promotion of ‘education for peace as a transversal aspect of [the Spanish] government action' (Podemos 2019). For FN/RN, the protection of fundamental freedoms of French and other non-specified European nationals contributes to shaping their overall conception of France’s international orientation. For example, their project for Europe is claimed to be ‘based on freedom, this founding value of our civilization born from Athens and Rome, this humanist value that Europe has taught the world’ (Rassemblement National 2019b, p. 2), while the French people should remember the long struggle for freedom of opinion, which they led and which they cannot give up without betraying (idem, 28). Eventually, therefore, liberal values are transversal to the moral foreign policy conception of all three parties, but differ in the way in which these values are presented to protect different constructions of who the people are.

The three parties also stress the need to allow their countries to play a more assertive role in the field of public aid for development, perceived as a *moral* responsibility (Le Pen 2017a; Le Pen et al. 2018, 9; M5S 2018a; Podemos 2016, p. 18). Also in this case, however, the way in which the three parties articulate this responsibility via the people/other dichotomy varies.

The ‘developer’ NRC of FN/RN is principally oriented toward Africa and driven by exclusionary considerations mainly towards migrants and Muslim people. According to the FN/RN, France must ‘be able to play a full role in co-development and advance a more ambitious policy, making aid conditional on close cooperation to control migratory flows to Europe’ (Le Pen et al. 2018, 12; Rassemblement National 2019a, p. 10). In fact, the articulation of this NRC for FN/RN is two-pronged. On one hand, it is based on aid for the development of schools, agricultural systems, and aid to strengthen defence and security tools from African countries. On the other hand, France should responsibly take strong diplomatic action ‘alongside its international partners and in close collaboration with the countries concerned’, to establish 'provisional humanitarian camps under UN control in these countries and ensure their protection, including by sending military forces’ (Le Pen et al. 2018, p. 10). Falk and Stahl (2022) claim that conceptualizing official development assistance as antimigration and antiterrorism policy was, in fact, the unique and rare selling points of FN/RN in this area, ‘void of idealistic goals' such as those advanced in the 2030 Agenda for sustainable development, where no one shall be left behind.

The ‘developer’ NRC of *Podemos* is as assertive as that of FN/RN, but follows a completely different logic. This role is conceived in inclusionary terms and is consistent with internationally agreed principles, namely with reference to the need for Spain to carry out a consistent and pragmatic implementation of the 2030 (Podemos 2019, p. 103). Commitment to sustainable development is key and is conceived as a form of participatory governance for the country. In general, the NRC of ‘developer’ is based on the idea of Spain as undertaking reforms and concrete measures to advance the coherence of all governmental actions with universal human rights-related principles (Real Instituto Elcano 2015, p. 36).

Development is one of the ‘5 stars’ on which the M5S was formally established in 2009. However, this theme does not surface in the official ‘*programmi esteri*’ of 2018, except for the point on disarmament as a prerequisite for peace which is explicitly linked to the promotion of the 2030 Agenda (Diodato 2022). Development is the basis for other chapters of the M5S manifesto, including that on ‘migration’. The available documents return a certain articulation of ‘developer’ for the Movement, caught between two logics. On one hand, they mark a more inclusionary approach based on promoting sustainable development and participation. The Italian development policy should be shaped by ‘direct democracy’, especially to face environmental challenges (Petrocelli 2016). On the other hand, M5S presents a more exclusionary conception of this role, which revolves around the stark opposition to employing public development aid funds for the integration of migrants in Italy, otherwise ‘this policy will continue to represent only an encouragement to get on the rafts’ (Spadoni 2017). Similarly to the FN/RN, the idea of development conceived by the M5S is based on co-responsibility and an effort to stem migratory flows to Europe. After all, ‘the borders of Italy are the borders of Europe’ (M5S 2019). As established when the Movement advanced a bill on the matter in Parliament in the mid-2010s, the underlying goal in this sector is to replace the idea of development as charity with one of actual cooperation, according to which interventions and programmes by Italy must primarily correspond to a request from the partner country (Spadoni 2014).

## Concluding remarks

This paper has investigated the foreign policy orientations and tasks conceived by three populist parties in Europe that are located at different points of the inclusionary/exclusionary continuum. Integrating the NRC framework to current scholarship on this matter, it has sought to set the stage for an original approach to delve into the amply demonstrated heterogeneity of populist foreign policies in Europe, and assess whether some convergence could be found among how diverse populist parties intend and present the broader role their countries should play internationally.

Focusing specifically on foreign policy discourse as expressed primarily through political manifestos, the analysis has shown that the heterogeneity of these parties’ foreign policies can in fact be understood also as the outcome of different discursive articulations of a common set of NRCs, which constitutes an underlying area of convergence among these. More specifically, the paper has unveiled a general role conception which underlies the foreign policy discourses of the three parties analysed: a principled commitment to lead the transformation of the current regional and international systems.

While the single NRCs assigned to these populist parties do not necessarily distinguish them from their non-populist national counterparts, their shared co-occurrence traces a common foreign policy contour which is distinctively populist from different conceptual angles. The co-occurrence of the ‘transformative agent’ role-subset shows that the foreign policy of these very diverse populist parties is, in fact, equally conceived through the lens of the ‘elite-underdog’ opposition, stressing antagonism between ‘us’ and ‘others’ and a desire to overturn the status quo to lead their people’s interest and protect it from a foreign other (see also Chryssogelos 2017; Wojczewski 2020; Giurlando 2020). The co-occurrence of the ‘value advocate’ role-subset recalls indications that populist ideology *tout court* constructs foreign policy as ‘restorative, trying to protect rights, values, and prosperity from external, corrupt and elite forces’ (Boucher and Thies 2020, 713). It also confirms that these parties often conceive and present their driving principles and values as the very basic motivation for conceiving a foreign policy in a world that is essentially depicted in highly moralistic terms (Destradi et al. 2021, p. 673). Furthermore, the co-occurrence of the second role-set suggests that these parties eventually intend to unify all individuals within their self-constructed understanding of ‘people’, making direct appeal to a series of principles, sovereignty *in primis*, which essentially replicate in foreign policy-making the function of the ‘general will’.

The type of pursued international and regional transformation, and the values on which this is to be achieved, however, change according to how exclusionary/inclusionary each of these parties constructs and presents the people/other dichotomy in their foreign policy discourse. *Podemos*, presents a strongly inclusionary idea of the change it would lead. It provides a broad understanding of who the people are in their conception of international affairs, which occasionally even reaches a universalist scope. Values and principles that shape *Podemos* role in foreign policy discourse are generally conceptualized as universalist too. The ‘other’ is only constructed vertically and is made up of those national and international institutions, norms and elites that threaten or reduce popular sovereignty, in Spain and in every corner of the world, and the rights and freedoms that all people should have recognized and protected.

The FN/FR aims to lead a regional and international transformation which is primarily constructed and presented in exclusionary economic and ethnic/cultural terms, both vertically, towards national and international elite and horizontally, towards migrants and religious groups. With regards to Europe, indeed, those who do not ‘wish to adopt […] minimum standards of common culture will have to make a choice between complying with them or changing cultural area’ (Le Pen et al. 2018). In fact, the FN/RN conception of ‘the people’, especially if one looks at the European manifesto, appears more ‘selective’ than exclusionary *tout court*. Their conception of France is that of a *primus inter pares*, inspiring and leading a revolution against current European and international institutions based on refusal of ‘imported multiculturalism’ (Rassemblement National 2019b, p. 57) and the promotion of liberal values which are shared with a vaguely defined group of European peoples—not by a European people, though—and nations characterized by common geographical, historical, and civilizational characteristics (Rassemblement National 2019a, p. 9; 2019b).

Finally, although the M5S shows elements of both in its general foreign policy discourse, it appears to move more in the FN/RN track, protecting vertically and horizontally a vague and narrowly defined ‘people’—the Italians, or the south-Europeans—against a quite diversified ‘other’, made of supranational financial institutions, European and national politicians and officials, international political and military organizations, and migrants. The idea of transformation ahead advanced by the Movement is therefore not clear (Diodato 2022), and the values on which its conception of foreign policy is based remains ambivalent, although its militants seem to privilege those points of the agenda that favour exclusion rather than inclusion (M5S 2018a).

The area of convergence that underlies these differences has been identified among the NRCs of three carefully selected populist parties in a circumscribed geographic area. Case-selection was meant to capture the rationale underlying the diversity of populist expressions in Europe. However, populist ambiguity does not allow one to claim that the identified convergence in political discourse represents a common contour for all European populist parties, let alone for other regions.

However, the empirical findings and the conceptual apparatus that was built for this paper are believed to represent an original contribution to advance into this broader and more complex research puzzle. They represent a further building block to complement findings on other common aspects of populist rhetoric in international relations which were unveiled from recent studies, especially those focusing on national identity conceptions (Friedrichs 2022) and discursive strategies (Wojczewski 2020), and present a fruitful application of the inclusionary/exclusionary classification beyond domestic politics. In addition, they provide an analytical tool that may help refine existing findings on the effects of these parties on national foreign policy-making and implementation where populists have reached a position of power in their respective countries.

Efforts to consolidate this contribution would entail expanding the empirical scope of analysis, both considering the NRCs of other relevant parties representing other positions across the exclusionary/inclusionary spectrum, and widening the time frame in reference. This would allow assessing whether and to what extent the common contour identified in the foreign policy discourse of FN/RN, Podemos and M5S is consistent and could apply as such to the entire heterogeneous and contradictory galaxy of European populism, also with the view to understand whether there is a ‘populist challenge’ to traditional foreign policy in the continent, and to favour a comparison with populist foreign policy in other regions.
